# Effectiveness of Spirituality-centered Cognitive Therapy and Contextual Transdiagnostic Approach on marital mutuality, dissociative experiences, and cancer-related fatigue in women after mastectomy

**DOI:** 10.1192/j.eurpsy.2025.1785

**Published:** 2025-08-26

**Authors:** M. Firoozi, M. Sharifi, O. Esmati, Z. Kiek, C. A. Lewis

**Affiliations:** 1 Psychology, University of Tehran, Tehran; 2 Psychology, Islamic Azad University, Bandargaz Branch, Bandargaz; 3 Psychology, Islamic Azad University, Azadshahr Branch, Azadshahr, Iran, Islamic Republic Of; 4 Psychology, Leeds Trinity University, Leeds, United Kingdom

## Abstract

**Introduction:**

Breast cancer remains the most frequently diagnosed cancer and a leading cause of cancer-related deaths in women worldwide. Post-mastectomy, women often experience heightened emotional distress, diminished quality of life, and interpersonal challenges.

**Objectives:**

This study seeks to evaluate the comparative effectiveness of Spirituality-Centered Cognitive Therapy (SCT) and the Contextual Transdiagnostic Approach (CTA) on marital mutuality, dissociative experiences, and cancer-related fatigue in women after mastectomy.

**Methods:**

In this randomized controlled trial, 132 women, selected based on inclusion criteria from an initial pool of 167, were randomly assigned to two intervention groups: SCT (n=66) and CTA (n=66). Each group underwent eight 60-minute therapeutic sessions over four weeks, held twice weekly from August to September 2022. Standardized questionnaires assessing marital mutuality, dissociative experiences, and cancer-related fatigue were administered pre- and post-intervention. Multivariate analysis of variance (MANOVA) with repeated measures was employed for data analysis using SPSS-24.

**Results:**

CTA demonstrated superior effectiveness in reducing dissociative experiences compared to SCT. Conversely, reductions in cancer-related fatigue were more pronounced in the SCT group. Improvements in marital mutuality were comparable between the two therapeutic approaches.

**Conclusions:**

Both SCT and CTA proved effective in enhancing psychological and relational well-being in women post-mastectomy. However, SCT more effectively alleviated cancer-related fatigue, while CTA showed greater efficacy in addressing dissociative experiences. These findings highlight the potential of integrating tailored therapeutic approaches to improve quality of life for breast cancer survivors.

**Disclosure of Interest:**

None Declared

